# Security Risks and User Perception towards Adopting Wearable Internet of Medical Things

**DOI:** 10.3390/ijerph20085519

**Published:** 2023-04-14

**Authors:** Sanjit Thapa, Abubakar Bello, Alana Maurushat, Farnaz Farid

**Affiliations:** School of Social Sciences, Western Sydney University, Penrith, NSW 2751, Australia

**Keywords:** wearable medical devices, IoT, cybersecurity, healthcare

## Abstract

The Wearable Internet of Medical Things (WIoMT) is a collective term for all wearable medical devices connected to the internet to facilitate the collection and sharing of health data such as blood pressure, heart rate, oxygen level, and more. Standard wearable devices include smartwatches and fitness bands. This evolving phenomenon due to the IoT has become prevalent in managing health and poses severe security and privacy risks to personal information. For better implementation, performance, adoption, and secured wearable medical devices, observing users’ perception is crucial. This study examined users’ perspectives of trust in the WIoMT while also exploring the associated security risks. Data analysed from 189 participants indicated a significant variance (R^2^ = 0.553) on intention to use WIoMT devices, which was determined by the significant predictors (95% Confidence Interval; *p* < 0.05) perceived usefulness, perceived ease of use, and perceived security and privacy. These were found to have important consequences, with WIoMT users intending to use the devices based on the trust factors of usefulness, easy to use, and security and privacy features. Further outcomes of the study identified how users’ security matters while adopting the WIoMT and provided implications for the healthcare industry to ensure regulated devices that secure confidential data.

## 1. Introduction

The advent of mobile computing and other cutting-edge technologies has paved the way for health monitoring through ubiquitous devices connected to the internet. These devices, commonly called the Wearable Internet of Medical Things (WIoMT), are now essential for monitoring various medical and health risks. With the capability to track changes to the body, such as monitoring sleep, heart rates, calories burned, and distance travelled, they have become an indispensable tool for healthcare professionals and individuals alike [1].

The authors in [2] described the WIoMT as interconnected individual medical devices that communicate over a network to enable data collection and sharing. The most commonly used WIoMT are Wearable Fitness Trackers such as fitness bands, patches, and smartwatches with health monitoring functions. Other forms include Wearable ECG Monitors, Wearable Blood Pressure Monitors, Biosensors, Smart Patches and Ingestible Sensors. Wearable Fitness Trackers monitor steps, heart rates, sleep hours, and burnt calories [3]. Likewise, Wearable ECG Monitors gather and share ECG data using Wi-Fi [4]. Wearable Blood Pressure Monitors are non-invasive and cuffless devices measuring blood pressure [5]. Biosensors detect specific analytes in a biological sample, and Smart Patches monitor various physiological measures such as pulse [6,7]. Ingestible Sensors measure temperature, pressure, and other chemical and physical parameters [8].

The WIoMT evolved from the Internet of Things (IoT) and the Internet of Medical Things (IoMT). The Internet of Things (IoT) is an encompassing term for all devices connected to the internet, such as devices from simple sensors to smartphones [9]. They are further described as interconnected devices that communicate over the internet to enable data collection and sharing [9]. They have been widely used in various areas such as personal homes, the medical sector, automation for industry and manufacturers, smart environments, traffic management, and several more. The IoMT is a binding domain under the IoT [10]. It is a group of health devices and products that connect with healthcare systems via networks [2]. Medical devices accessing Wi-Fi allow machine-to-machine networking, which forms the basis of establishing the IoMT [11]. The IoMT comprises several crucial aspects, such as actively monitoring patients with long-term or chronic conditions, recording medical attention requests and hospitalised patients’ status, and wearable patient care devices that transmit data to hospitals [11]. Health devices that can be converted or integrated into IoMT technology include infusion pumps connected to analytics supports and hospital beds equipped with devices that monitor patients’ vital signs [11]. Similarly, [12] also define the use of the IoT in healthcare for health assistance as the IoMT. This refers to the integration of the IoT with medical devices that enable improved patient comfort, cost-effective medical solutions, faster hospital treatments, and even more personalised healthcare. These technologies facilitate the diagnosis of certain diseases, mainly without the need for a healthcare professional [13].

As the WIoMT industry advances, so do concerns about security and privacy [14,15]. These devices pose significant security and privacy risks as they continuously collect users’ personal health information in real time, which is considered more vulnerable than other types of data [16]. The decision to adopt wearable medical devices involves a risk analysis that takes into account the trade-off between significant benefits and perceived security threats since using them requires the disclosure of personal medical information [17]. Security and privacy issues are crucial in influencing users’ adoption and trust of medical devices. Thus, there is a need to investigate users’ perceptions of security and trust concerning the WIoMT. This study aims to explore the security and privacy risks associated with the WIoMT and understand the factors influencing users’ trust decisions when adopting the technology. The study is guided by two research questions: (a) What are the factors that affect the trust and adoption of the WIoMT? (b) What are the security risks associated with using the WIoMT? The findings revealed that two dimensions and five domains predicted consumers’ intention to use WIoMT devices. The two dimensions were product-related and security-related factors, while the five domains included functionality, reliability, perceived usefulness, perceived ease of use, and perceived security and privacy. The study established that these factors were significantly associated with the intention to use WIoMT devices. Notably, perceived security and privacy was the most significant predictor of the dependent variable, intention to use.

To this end, the research questions and hypotheses of the study are discussed below, focusing on each of the factors and variables that were examined. The paper is structured as follows: Section 2 discusses the architectural context of the WIoMT, focusing on the theoretical framework and hypothesis development for this study. Section 3 outlines the detailed methodology used in this research. Section 4 presents and discusses the results of this study, followed by a comprehensive discussion of the findings in Section 5. Finally, Section 6 provides concluding remarks and highlights directions for future research.

## 2. Architectural Context of WIoMT

In traditional medical scenarios, healthcare professionals collect and manage patients’ health data manually using standard medical equipment, such as stethoscopes, thermometers, and blood pressure monitors. Health data are often recorded on paper or in electronic health records (EHRs). In some cases, these data are not accessible via the internet or other digital networks.

In contrast, medical IoT scenarios involve using connected devices, such as medical sensors, wearables, and implantable devices, to collect and transmit real-time patient health data to healthcare providers. These data can be used to monitor patients remotely, provide more personalised care, and detect health issues early on. Medical IoT devices can also be integrated with electronic health records (EHRs) to provide a more comprehensive view of a patient’s health status, medical history, and treatment plan.

In contrast to traditional medical scenarios, medical IoT scenarios involve a higher degree of connectivity and automation in collecting and managing health data. Medical IoT scenarios leverage advanced technologies to provide real-time monitoring, more personalised care, and improved accuracy and efficiency in managing patient data. However, the use of medical IoT devices also raises concerns about security, privacy, and data protection, which must be addressed to ensure the safety and well-being of patients.

**Figure 1:**
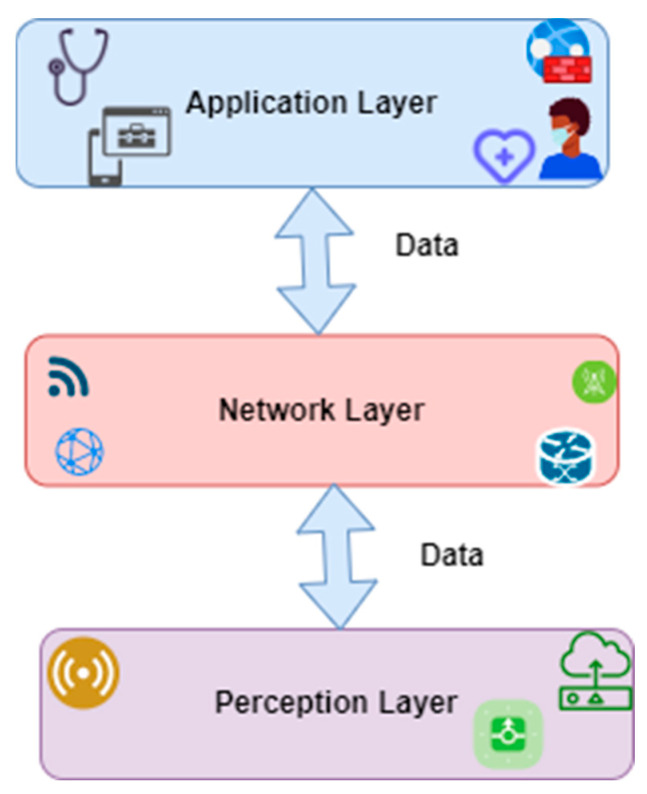
Fundamental architecture of WIoMT [18].

The architectural basis of the WIoMT is built on three different layers, as illustrated in Figure 1. The application layer includes clouds and servers where the device from the perception layer is connected through the network layer and stored in an abstract space and is easily retrieved when required or necessary. The network layer is the component commonly known as routers and gateways. So, they act as a bridge between the application and the perception layers. The perception layer is ‘the tangible things’ that ‘users’ use. The perception layer consists of various devices and instruments with sensors and actuators. The WIoMT follows a very similar architectural model to the IoT, where sensors, RFID, and smartphones are connected through various networks and are operated from multiple application domains. Although the structure is similar, the difference lies in the perception layer of devices, as the IoMT perception accumulates medical information with several devices [10].

Healthcare service providers are growing their medical practices to include the Wearable IoMT, which has become increasingly crucial as an efficient method to monitor patients’ health [19]. These devices have given rise to new architectures, applications, and standards related to addressing current health challenges and have further enhanced the development of the individualised and single-user-based WIoMT [20]. Some real examples of WIoMT devices are provided in Table 1 below:

WIoMT devices have become significant in providing different benefits to the medical sector, such as minimising patients’ travel time and expenses, delivering medical care in places with limited accessibility, and enhancing the delivery of critical knowledge to health personnel from distant places. For instance, [21] observed that the beneficial use of WIoMT devices ensured the monitoring and consciousness of individual health statuses among users. [11] also claimed that the modern WIoMT promises personalised and enhanced healthcare services, such as portable medical apps that are shifting hospitals’ reach from domestic to international healthcare monitoring agencies. Such wearable applications, enabled by Bluetooth and close-field connectivity, change the precautionary medicine domain and the lives of individuals with chronic disease by providing continuous health surveillance, detection, and better care. Nonetheless, despite these benefits of the WIoMT, specific challenges and risks exist that must be considered before adoption.

### 2.1. WIoMT Adoption Challenges and Risks

Healthcare wearables should be considered high-privacy- and high-security-risk gadgets since they continuously collect users’ personal health information in real time. Personal health information is more susceptible than other forms of data, such as demographic or primary consumer data [16]. The choice to embrace such technology requires a security and privacy assessment that weighs the trade-offs between significant benefits and perceived security/privacy hazards, because the use of such devices involves the exposure of personal medical data [17]. When a user believes that the anticipated advantages of using a wearable system outweigh the risks, the user will usually prefer to use wearable healthcare technology. In the past, society has concentrated on the negative consequences of sharing personal data, such as security flaws, forgeries, and data theft. The authors in [22] carried out a preliminary investigation to see if individuals considered the costs and advantages of possibly surrendering some privacy in exchange for using electronic health records (EHRs). They found a substantial need for research on evaluating the effect of the user’s perspective whenever technology is used to exchange personal information [22]. Healthcare wearables are typically conceived as consumer items, which may restrict their utility as health apps as they are easily accessible and available to everyone who may not have bought them for health management in the first place [23]. Additionally, they have the potential to alter healthcare delivery methods and the acquisition and transmission of sensitive personal information. Therefore, these devices tend to complicate matters related to privacy, security, sharing, autonomy, permission, ownership, access, and data valuation [24], as illustrated in Table 2.

One of the major security and privacy issues in the WIoMT is clear text login information and clear text HTTP data processing [25]. This implies that the login and passwords of these devices are recorded in log files as plaintext [25]. The data shared among different domains are sent as plain text, and no security measures such as encryption are used while transmitting the data. In a study by [26], it was found that sensor tracking is the most commonly identified threat in the perception layer of WIoMT. Additionally, tag cloning, side channel, physical harm, and jamming threats were identified as potentially significant threats in the perception layer [26]. Further complications arise with preserving the security and privacy of sensitive data since unauthorised access to the medical data of users has been reported quite often. These security and privacy issues could result in the deterioration of the effectiveness of WIoMT and adversely impact individuals’ sensitive health information [26]. The other prominent issues with WIoMT are technical safety issues which occur due to the high dependence on device operating systems [27].

WIoMT devices are also susceptible to cyber attacks since cybercriminals can target devices that usually have less security protection. For example, MyFitness Pal was hacked in 2018, exposing the data of up to 150 million users, which were subsequently sold on the dark web.

Recently, it was found that 94% of healthcare organisations have been victims of cyber attacks on medical devices and infrastructure [28]. The resource-constrained nature of the IoMT can be exploited by malicious actors, who may use it to carry out severe cyber security attacks against the network infrastructure [29]. Although the international standards community has taken a lead role in developing and modifying existing standards to address issues of malware infections, vulnerabilities and cyber attacks, the proprietary nature of previously non-interoperable medical devices limits integration between vendors’ products [30,31]. This results to errors in communication since interoperability and integration do not equate.

Similarly, the Food and Drug Administration (FDA) has issued two guideline publications on the management of cybersecurity in medical devices. Manufacturers should integrate risk management during the development of medical devices and furnish the FDA with risk data when submitting products for clearance. However, the FDA’s guidelines do not evaluate the risk assessment method used by manufacturers to assess cyber threats, nor do they give criteria for manufacturers to determine possible countermeasures [32]. Though such recommendations are non-binding, they acknowledge that the shift in the operating environment of the WIoMT needs urgent attention [32]. A debate also exists over the definition of medical devices and under what kind of circumstances any product shall be called a medical device [32].

### 2.2. Theoretical Framework and Hypothesis Development

Trust and security risks play a vital role in determining WIoMT adoption. A study conducted on 489 IoT users by [33] exposed that perceived usefulness and enjoyment significantly affect behavioural intention through the perceived value and trust of IoT services. Similarly, it was also found that IoT adoption is determined substantially by perceived privacy risk [33]. In another study, some major factors influencing and acting as significant predictors of adopting IoT are performance expectancy, effort expectancy, social influence, hedonic motivation, and price value [34]. According to Dong et al., many researchers have used experience theory and TAM to explore users’ general perceptions through IoT. In contrast, the finding that users embraced IoT systems through healthcare is constrained [35]. Hence, there is a need to explore users’ security and perception of the WIoMT.

Ref [36] classified trust-related findings into three categories based on their different theories:

1. Trust is defined in personality theory as a belief found in conduct, which emerges early in the personality’s psychological growth.
2. Trust is defined in sociology and economics as a process that occurs inside and between communities, organisations, and individuals who trust them.
3. In social psychology, trust is defined as the intentions and desires of the innocent party in a transaction, the concerns arising from that transaction and the various components that aid or obstruct the creation and maintenance of that trust.

**Figure 2:**
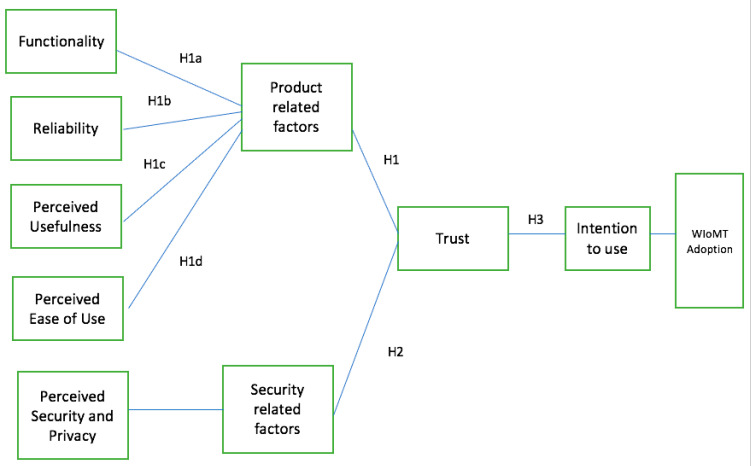
Conceptual Model.

This study adopts a social–psychological viewpoint for examining variables impacting users’ trust and intentions in using the WIoMT as it focuses on transactional issues and risks associated with the WIoMT. To better understand the significance of the trust factors on the adoption of the WIoMT, a conceptual model (Figure 2) has been proposed that draws from the diverse understanding of trust and is theoretically based on the Technology Acceptance Model (TAM). The TAM is a prominent theory that explains how a user adopts and uses technology. The TAM has been examined and validated by researchers, and it has been proven acceptable as a theoretical underpinning for technological adoption [37]. There are numerous theoretical foundations for technology adoption, but according to the TAM, various variables influence users’ decisions about how they will use a newly given system. The two most important criteria in behavioural intention to use technology are perceived ease of use and perceived utility [38].

WIoMT devices should have higher adoption rates to simplify usage for consumers. Additionally, the acceptance of WIoMT devices must be analysed from the users’ perspectives. Therefore, the following research questions and hypotheses are considered in this study:

A. What factors influence the trust and adoption of the WIoMT?
B. What are the security risks associated with adopting the WIoMT?

Numerous quantifiable and non-quantifiable variables impact the trust of the WIoMT. The conceptual model classified the variables under two main dimensions: product-related and security-related. Each dimension consists of several variables.

#### 2.2.1. Product-Related Factors

Several product-specific factors may affect users’ decisions to trust a WIoMT product. Various models and studies have suggested various factors affecting confidence that influence the adoption decision. These are:

**Functionality and reliability:** This refers to whether technology has the ability or capacity to accomplish a specified task by having the required characteristics and will function correctly and reliably in a consistent manner [39].

Trust in the functionality of a technology depends on the capacity of that technology to perform properly. It is noted that users’ trust is based on the perception that the service or product will carry out its expected and requested function [40]. Because errors are not acceptable to users of any technology, there is a significant impact of the absence of errors on trust toward WIoMT adoption, similar to IoT devices [41]. Hence, the following hypothesis are considered:

**H1a:**  *The functionality of WIoMT devices has an effect on trust towards WIoMT adoption.*

**H1b:**  *The reliability of WIoMT devices has an effect on trust towards WIoMT adoption.*

**Perceived Usefulness:** This is defined as the degree to which one believes that using the technology will enhance their performance [39].

Numerous studies have demonstrated the positive relationship between IoT products or services adoption rates and the user’s perception of the products or services facilitating their everyday lives. Therefore, the perceived usefulness of the *WIoMT* devices must be advocated to achieve successful adoption.

**H1c:**  *Perceived usefulness has an impact on trusting WIoMT devices.*

**Ease of Use:** This refers to the degree to which one believes that using the technology will be effort-free. According to the ease of use, technology plays a significant role in building up consumers’ trust towards a technology.

**H1d:**  *Perceived ease of use has an impact on trusting WIoMT devices.*

#### 2.2.2. Security-Related Factors

In the WIoMT context, security indicates the degree to which a person believes it will be risk-free to use a WIoMT product. When introducing new technology, security is a significant user concern and directly affects the trust of the user of a particular product or service and, thus, the acceptance of technology [42].

**Perceived Security and Privacy:** This factor is concerned with the ability of the trustee to achieve significant security goals such as confidentiality, which assures that only authorised users can have access to sensitive data; availability, which guarantees the resilience of systems even though attacks occur; integrity to ensure the protection of original forms of data; and authenticity, which eases any interaction between confirmed devices. Security is always a critical issue with which users are concerned regarding trust towards adoption. The levels of security and privacy are critical characteristics of IoT technology that affect the development of users’ confidence in them, as they assure users that they will be safe [40].

According to [43], users tend to trust IoT devices that use credible entities for authentication and access control. Such devices that show the ability and willingness to protect themselves are noted as trusted devices. Therefore, it can be deduced that there is a positive relationship between trust and a WIoMT product’s security level.

**H2:**  *Perceived security and privacy have an impact on trusting WIoMT devices.*

Trust may also be considered as a significant factor affecting the behavioural intention to use any IoT technology [44]. Therefore, trust plays a significant role in users’ perception and adoption of WIoMT.

**H2a:**  *Perceived security and privacy have an impact on trusting WIoMT devices.*

**H3:**  *Trust has a direct impact on behavioural intention to use WIoMT devices.*

The null hypotheses of all the above-hypothesised statements (H1 to H3) are also vital in the sense that they will still indicate some Hlevel of WIoMT adoption concerns due to security risks in the event that H1 to H3 are non-significant. The methodology by which these hypotheses are explored is discussed in the next section.

## 3. Methodology

This study adopted quantitative methods using a non-experimental cross-sectional design in a questionnaire. The reason for employing a questionnaire is so that we could benefit from insights and perceptions that allow for correlation and regression analysis to be employed. The questionnaire consisted of two sections. The first section used demographic information to segment data and compare respondents. This section collected information regarding age group, gender, and level of technical proficiency in using computers. The objective of the second section was to assess the factors affecting trust based on the conceptual model in Figure 2. For the validity and reliability of the questionnaire, the questions were adapted from existing survey instruments and the WIoMT risks and control gaps identified from the literature. The IoT is an extensively researched area that allows for the acceptance of previously used scales. Although the scales were created precisely to address specific IoT challenges, these could be adapted to address the identification and understanding of security risks and users’ perception of WIoMT adoption. The questionnaire mainly represented two dimensions of factors with a hypothesis set for each factor.

### Participants and Procedure

This study included 189 participants that belonged to diverse cultures, backgrounds, and age groups. The applicable and effective number of participants was confirmed by implementing the rule set by Tabachnick and Fidell, which is N ≥ 50 + 8 m, where m is the number of predictors. Similarly, [45] states that 15 participants per predictor is appropriate for sampling. This study meets the assumptions for both, with 189 participants from whom primary data were collected. The survey included demographics, perceived usefulness, perceived ease of use, functionality and reliability, perceived security and privacy, and behavioural intention to use. All ethical approvals necessary for the project were considered and obtained from the Human Research Ethics committee. In addition, a project description, participant information sheet, consent forms, and recruitment documents were developed. The survey was conducted online, with participants signing up for the study, and data were collected via Qualtrics (an online-based survey platform). After participants accessed the survey, they were required to read the participant information sheet that gave a basic summary of the study and outlined what participants would be expected to do. Participants were then required to sign the consent forms.

The collected data were exported to Microsoft Excel from Qualtrics for cleaning, screening, and coding. They were further exported to Statistical Package for Social Science (SPSS, Version 21, IBM, Sydney, Australia) for statistical analyses. At first, for descriptive statistics, the frequency, percentage, mean, median, and standard deviation were calculated as appropriate. The frequency and percentage were calculated for all the categorical variables, such as age groups, gender, technical proficiency in using computers, perceived significance of technology, and each domain’s statement. The mean, median, and standard deviation were calculated for all the numerical variables, such as age and scores for each domain and dimension. Graphical and tabular presentations were used where needed.

For inferential statistics, correlations were tested between domains and dimensions at a 95% Confidence Interval with a *p*-value less than 0.05 being statistically significant. Pearson Correlation analysis was utilised when the association between two numerical variables was to be determined if they were non-normal variables [46]. The study calculated scores for all domains based on their ordinal categories. These scores were correlated in pairs for each respondent to determine a significant association between two domains in the pair.

Additionally, regression analysis was conducted to determine the factor that had the most influence on the outcome variable of this study. Additionally, regression analysis calculated the percentage variation of the outcome variable (behavioural intention to use) represented by the independent/input variables (perceived usefulness, perceived ease of use, functionality and reliability, and perceived security and privacy). The regression model generated was also tested to determine if it predicted the outcome variable, and if it did, to also determine which variable had the highest power to predict the outcome variable.

According to [47], a correlation analysis with a series of regression analyses between variables can display significant outcomes and precisely find factors that influence consumers’ decision to accept WIoMT devices. IBM SPSS software for statistical analysis was used to analyse the data in this study. Based on correlation analysis, it was expected that there might be a correlation between security and perceived usefulness towards trust and a correlation of intention to use that leads to attitude. The regression analysis was expected to find the strongest relationship between factors leading towards trust and how they affect users’ perceptions.

## 4. Results

This study was conducted using a survey (via Qualtrics) with 55 questions, initially involving 243 participants. The data screening revealed 32 blank surveys and 1 case of ineligible participant, which were removed before exporting to Microsoft Excel and SPSS for data cleaning and analysis. A missing value analysis in SPSS showed 21 cases had incomplete responses with more than 50% questions unanswered, and the remainder had at least 1 question unanswered. The former 21 cases were deleted as per the suggestion by [48], and for the latter ones, regression imputation was conducted to replace the missing data with estimated values. Ultimately, the study included 189 responses. The analyses based on the responses provided by the participants were conducted through SPSS software, version 21, with the following outputs noted as findings/result.

### 4.1. Demographics

The first set of questions was related to demographics and other related information. Starting with age, more than half (42.9%) belonged to the 25 to 34 years age group, followed by 37% of those between ages 18 and 24, 9.5% between 35 and 44, and 6.9% between 45 and 54. There were seven respondents aged 55 or above (Table 3). A little more than half of the respondents identified themselves as female. Males comprised 46.6% of the total respondents.

When respondents were asked about their technical proficiency in using computers, almost half (45%) indicated they were at the “Somewhat Above Average” level, 40.2% at the “Average” level, and 13.8% at the “Far Above Average” level of technical proficiency. Only 1.1% stated they had a “Somewhat Below Average” level of technical proficiency. Likewise, when asked about technology having great significance in their lives, the majority (88.4%) affirmed with “Definitely Yes”, 11.1% answered with “Probably Yes”, and only 0.5% gave the answer “Might or might not”.

Additionally, more than half (65.6%) of them responded they were familiar with WIoMT devices, and among them, 77 mentioned they used such devices, and 47 said they did not. Similarly, when those who used such devices were asked if they were aware of any of the listed WIoMT devices, only one-fourth (23.8%) selected Wearable Fitness Trackers, 3.2% selected Wearable ECG Monitors, 6.9% Wearable Blood Pressure Monitors, 2.1% Smart Patches, and 4.8% mentioned they were aware of none of the listed devices.

### 4.2. Security Risks

There were six statements related to security risks within the perceived security and privacy domain. The theme of the questions was unauthorised access to data; malware infections and vulnerabilities; lack of regulation and compliance; unsecured network connectivity; lack of encryption; and lack of patching and device updates. (Table 4). Taking a descriptive look at each risk, for unauthorised access to data, more than two-thirds of the respondents agreed on its effect on the WIoMT, while 23.2% gave a neutral reply, and only 6.4% denied its effect on the performance of such technologies. Similarly, for all the other security risks, more than sixty percent of the respondents affirmed the effect of those risks on WIoMT devices. Only less than 10% of the respondents felt these risks did not affect the performance of WIoMT devices.

If we observe the proportions of participants responding to the statements related to security risks, most of them felt “unauthorised access to data” was the most prominent risk for these devices, followed by “unsecured network connectivity” and “malware infections and vulnerabilities”.

### 4.3. Reliability and Validity

Cronbach’s alpha value was used to test the internal consistency within each domain and dimension. Among the domains, the internal consistency was calculated for perceived usefulness, perceived ease of use, functionality and reliability, perceived security and privacy, and intention to use due to the multiple number of questions in each. It was found that the internal consistency in all five domains (perceived usefulness, perceived ease of use, functionality and reliability, perceived security and privacy, and intention to use) was higher than the acceptable value of 0.7 as demonstrated in Table 5, thus indicating a reasonable and acceptable level of reliability and validity as recommended by [41].

Among three dimensions, internal consistency was calculated for product-related factors and security-related factors, and the internal consistency for both dimensions was in the acceptable range (i.e., above 0.7) (Table 6).

### 4.4. Correlation

a. Correlation between Product and Security-related factors

i. Functionality vs. Perceived Security and Privacy

There was a weak positive correlation between functionality and perceived security and privacy, and it was significant at *p* < 0.001 (Table 7). This means that, with the increase in the functionality of WIoMT devices, there is a significant increase in the perceived security and privacy of such devices among the consumers.

i. Reliability vs. Perceived Security and Privacy

There was a moderate positive correlation between reliability and perceived security and privacy, and it was significant at *p* < 0.001 (Table 8). This means that, with the increase in the reliability of WIoMT devices, there is a significant increase in how consumers perceive the security and privacy of such devices.

i. Perceived Usefulness vs. Perceived Security and Privacy

There was a moderate positive correlation between perceived usefulness and perceived security and privacy, and it was significant at *p* < 0.001 (Table 9). This means that, with the increase in the perceived usefulness of WIoMT devices, there is a significant increase in the security and privacy perception of such devices among consumers.

i. Perceived Ease of Use vs. Perceived Security and Privacy

There was a moderate positive correlation between perceived ease of use and perceived security and privacy, and it was significant at *p* < 0.001. (Table 10). This means that, with the increase in the perceived ease of use of WIoMT devices, there is a significant increase in the perceived security and privacy of such devices among consumers.

a. Correlation between Product and Security-related factors, and Intention to Use

The correlation analysis showed that there was a significant correlation between product- and security-related factors and intention to use (Table 11).

A moderate positive correlation could be observed between perceived usefulness, perceived ease of use, and perceived security and privacy with intention to use WIoMT devices, respectively. Additionally, there was a weak yet positive correlation between the functionality, reliability, and security risk of such devices with their intention to use. According to Table 11, all factors were found to be significantly associated with intention to use at *p* < 0.001 level. The correlation analysis indicated that all product- and security-related factors had a positive influence on the intention to use WIoMT devices, which led to the rejection of their null hypotheses and affirmation of their hypotheses.

As it was found that the functionality of WIoMT devices was significantly linked to use; H1a, “the functionality of WIoMT devices has an effect on trust towards WIoMT adoption”, was supported by this study. It means that the respondents were ready to adopt WIoMT devices provided that the devices have proper functionality. An increase in functionality was found to increase users’ perception of security and privacy. This creates more awareness of the potential risks of the use of WIoMT. It leads to trust, which finally results in the high possibility of adopting WIoMT devices, as intention to use becomes strong.

H1b states “the reliability of WIoMT devices has an effect on trust towards WIoMT adoption”. Reliability and intention to use were found to have a positive correlation in this study. The reliability of the WIoMT devices builds trust and gives way to an intention to use, resulting in the adoption of the WIoMT. This shows that despite lacking knowledge about device reliability, users are still willing to trust and adopt WIoMT devices.

H1c, “perceived usefulness has an impact on trusting WIoMT devices”, was also supported by the study. It was found to correlate with the intention to use positively. This proves that if the respondents understand the usefulness of the WIoMT, then they trust WIoMT devices.

“Perceived ease of use has an impact on trusting WIoMT devices” was indicated as H1d in this study. It was found to support this study as the perceived ease of use was significantly associated with the intention of use. It brings forth an insight that trust in the WIoMT and its adoption is highly impacted by perceived ease of use.

H2, “perceived security and privacy has an impact on trusting WIoMT devices”, was also supported by this study, as it was found to be significantly correlated with intention to use. Trusting WIoMT devices on these grounds ignited the intention to use among the respondents. Security and privacy always matter while trusting to adopt or use any WIoMT devices. It was found that the participants considered trusting WIoMT device companies and service providers in protecting their individual data. This hypothesis is further supported by the positive correlation of security risk and intention to use.

On the grounds of functionality, reliability, perceived usefulness, perceived ease of use, and perceived security and privacy, H3, “trust has a direct impact on behavioural intention to use WIoMT devices”, was found to be supported by this study.

All these variables were further explored in a multivariate analysis (regression) to determine the variables and factors with the strongest relationship and relevance when adopting WIoMT. The results are given below.

### 4.5. Regression

Regression was calculated and is shown in the tables below with the adjusted R square value showing 54% of variation in intention to use represented by reliability, perceived usefulness, perceived ease of use, functionality, and perceived security and privacy (Table 12). The value for the threshold of F was 0, which told us that the null hypotheses could be negated, and the research hypotheses could be accepted.

Furthermore, Table 13 shows how well the regression equation fit the data, and in this case, the model predicted the dependent variable significantly, i.e., the regression model statistically and significantly predicted the outcome variable.

Likewise, Table 14 shows the results of the multiple regression test. Here, the t-value of perceived security and privacy was the highest with significance at *p* < 0.001, and hence had the highest power to predict the outcome (intention to use WIoMT). This brings clarity that users depend on device security and privacy while trusting to adopt any WIoMT devices.

Based on power to predict, perceived security and privacy was followed by perceived ease of use and perceived usefulness in the prediction of intention to use WIoMT devices.

This implies that three of the considered independent variables (perceived usefulness, perceived ease of use, and perceived security and privacy) in this study were significant predictors of the dependent variable (intention to use). The following findings provide a summary of the regression results for each of the three domains:

1. Another significant aspect of WIoMT device adoption by the users found in this study was WIoMT devices’ security and privacy. It was found that WIoMT device adoption was affected by the perception of the user on this ground. It was found that if the user perception indicates the compromise of one’s privacy and security, the user is less likely to adopt WIoMT devices. Therefore, it became clear that concerns about privacy and security are of high consideration for users while trusting to adopt WIoMT devices.
2. In terms of evaluating the influence of users’ perceptions that using WIoMT technologies would make their lives easier and smarter, some enquiries were put forth. It was found that users who believe and understand the time- and effort-saving nature of WIoMT devices are influenced in their decision to adopt them. With respect to the questions regarding ease of use and device usefulness, the results indicate a positive attitude towards adoption if WIoMT devices are easy to use and able to demonstrate device usefulness. Perceived usefulness and ease of use are inter-related aspects and affect a user’s perception towards trusting to adopt any WIoMT devices.
3. Likewise, the regression analysis shows that the intention to use WIoMT devices is also determined by the user’s perceived security and privacy. When the user’s perceived security and perceived privacy are ensured by WIoMT, then the user gains trust in the device and intends to use the device.
4. This study was also geared towards investigating the significance of trust in affecting “behavioural intention to use” towards WIoMT adoption. It was found that trust generated in the user plays an important role in affecting the behavioural intention to adopt WIoMT. This implies that any user’s behavioural intention to use is influenced by trusting WIoMT devices.

## 5. Discussion

This study investigated the factors influencing trust and intention to use WIoMT devices. The study had two research questions: (a) What factors influence trust and adoption of WIoMT? and (b) what are the security risks associated with adopting WIoMT? The results confirmed that the two dimensions and five domains predict consumers’ intention to use WIoMT devices. These two dimensions were product-related factors and security-related factors. In contrast, the five domains were functionality, reliability, perceived usefulness, perceived ease of use, and perceived security and privacy. The study showed that these factors were significantly associated with the intention to use WIoMT devices. Perceived security and privacy was the most significant predictor of the dependent variable, intention to use. Each of the factors and variables studied in this research are discussed below with respect to the research questions and hypothesis of the study.

This study comprised individuals aged mostly between 25 to 34 years old, followed by those of 18 to 24 years of age, which is similar to the studies by [33,49,50,51,52]. This study took place in a university context where young adults comprise most of the population. In the other mentioned studies, online surveys were also utilised, mostly accessed by young people, not older individuals [53]. Similarly, there were more female respondents than males, which was opposite to the studies by [45,50,51]. However, this finding was similar to other studies by [33,52]. This may be explained by a study on the influence of gender on online survey participation, which showed that females are more likely to contribute to online surveys than males [45].

When asked about familiarity with WIoMT devices, in this study, more than half of the respondents mentioned they were familiar with such devices. Among them, 40.7% of the total sample had used them, which is quite different from the study by [49], which mentioned 81% of their sample had used at least one to five IoT devices, and from the studies by [33,51], in which 100% of the sample used IoT devices. This is an interesting finding for a sample extracted from a specific population belonging to a highly multicultural educational institution. It lets us predict the usage of such devices in the general population, and this information can be useful for promoters and marketers of such technology.

The reliability measure across the dimensions showed accuracy in the measurement to the extent that even if the respondents answered the questions multiple times, their pattern of answers remained the same every time. This was determined by Cronbach’s alpha higher than 0.7 for both dimensions. Similar values were also obtained in studies by [40,49]. Likewise, the reliability across the domains was also high, i.e., more than 0.7 for all domains. This is in coherence with other studies [49,50,51,54], where the reliability measure was above satisfactory for the concerned domains of the intention to wse WIoMT devices, such as perceived usefulness, perceived ease of use, perceived security and privacy, functionality, and reliability. There were similar results in a pilot study which incorporated the same dimensions and domains.

All product-related and security-related factors were found to be significantly associated with this study. They had a positive correlation, which means that with an increase in one, there was a subsequent and significant increase in the other. For example, if the functionality of WIoMT devices perceived by the consumer increases, then their perception of privacy and security of such devices also increases. Similarly, if these devices are perceived to be reliable, useful, and easy to use, their view regarding the privacy and security of using such devices also increases. This finding is also consistent with studies by [49,50].

Additionally, in this study, it was found that all product- and security-related factors were significantly and positively correlated with the dependent variable, intention to use. This result was similar to [50,54]. It illustrates that the theoretical framework for this study was connected with the findings derived from the analyses. In fact, it validates the hypotheses which stated that the product-related and security-related factors (perceived usefulness, perceived ease of use, perceived security and privacy, functionality, and reliability) all have an impact on trusting WIoMT devices, which in turn affect the intention to use them.

Similarly, the sub-domain security risk significantly correlated with the dependent variable, intention to use. In this study, the security risks identified fell into the following categories:

1. Unauthorised access to data;
2. Malware infections and vulnerabilities;
3. Lack of regulation and compliance;
4. Unsecured network connectivity;
5. Lack of encryption;
6. Lack of patching and device updates.

Most of the participants considered “unauthorised access to data” to be the most prominent security risk for WIoMT devices, followed by “unsecured network connectivity” and “malware infections and vulnerabilities”. There was a significant correlation with intention to use. When users perceive security risks in using the devices, their tendency to use such devices is affected, which is significant and not merely due to chance. According to the findings from this study, the security risk mentioned had an impact on the intention to use such devices. It showed that intention to use was affected when there is more agreement on the impact of security risk.

Furthermore, the regression analysis has shown all domains to predict factors of the dependent variable, the intention to use WIoMT devices. These domains are the independent variables: perceived usefulness, perceived ease of use, functionality, reliability, and perceived security and privacy. This is consistent with the studies by [33,49,50,51,55]. Among the predictors of intention to use, perceived security and privacy was found to have the highest power to predict the outcome. This finding was similar to the studies by [54,56], and the theory presented by [57]. It suggests that if users believe that the device protects their privacy and has security measures for their stored/shared information, they are more likely to use it. It is a basic expectation for any technology in the modern world, where people trust inanimate objects for their health and well-being. In other words, WIoMT service providers need to resolve security issues using various security controls such as encryption, data transparency and Public Key Infrastructure, and in general, by applying best data management practices. However, this finding was different to studies by [18,55], where other factors turned out to be more powerful predictors than perceived security and privacy. The conclusion is that product-related factors (mostly perceived usefulness) were the most influential predictor of intention to use WIoMT devices. It is also relevant because consumers buy a product only when they perceive something as useful. If a person does not see any need for it, even though it has the best security features and is very easy to use, he/she does not intend to use it, which in turn discourages him/her from buying it in the first place. However, our study showed that the privacy and security domain had the highest power to predict the intention to use such devices. In contrast, others, such as perceived ease of use and perceived usefulness, had lower power to predict in comparison. However, they were still the other predictors.

Overall, the results and findings indicated that the factors that influence the trust and adoption of the WIoMT were mainly perceived security and privacy, perceived ease of use, and perceived usefulness. Among these, perceived security and privacy had the highest power to predict the adoption of the WIoMT. All these predictors led the user to trust the devices and intend to use them. This means that users look for security features in such devices before trusting them. After they are assured of their ability to keep information private and secure, they look for ease in using them and how useful they are for themselves. Although there was a positive correlation between device functionality and reliability with the intention to use WIoMT devices, these became insignificant when adjusted with other factors. As for the security risks identified from the literature in this study, findings from the survey showed that risks of unauthorised access to data, malware infections, lack of regulation and compliance, unsecured network connectivity, lack of encryption, and lack of patching and device updates are significantly correlated with intention to use. This means they had a significant effect on the behavioural adoption of WIoMT technologies. These risks also comprised the sub-domain part of the security-related domain, the strongest predictor of the outcome variable.

### 5.1. Theoretical and Practical Implications

The proposed research model in this study predicted more than half of the outcome variables (R^2^ = 0.54). The significant variables that affect the intention to use WIoMT devices were perceived security and privacy, perceived ease of use, and perceived usefulness. Perceived security and privacy had the highest influence on the adoption of the WIoMT than other domains (ß = 0.691, t = 6.789). The implication of this outcome is that if the consumers of these technologies do not perceive the devices to store and/or relay information securely and privately, they will not have intention to use it in the first instance. This shows that WIoMT devices need to have the best security and privacy features to be relevant in the market.

Additionally, WIoMT technologies need to be easy to use so that users do not get discouraged from adopting these devices. After all, the devices are built to be used voluntarily. For many, the devices have not become necessities until and unless they have some health issues to manage on their own. Hence, these technologies need to be easily comprehensible for all age groups, especially older generations who more likely need to utilise of these wearables that track their health and help maintain wellness. Not only easiness in use, but WIoMT’s actual usefulness determines it utilisation among the users. They need to visualise what these devices can do to make their lives easier, and this can be done in various ways. One effective way can be to engage prominent people in the community who use these devices to advocate them to others. Hence, developers need to make these devices user-friendly and purposeful with exciting features to increase intention to use.

One of the most influential factors that leads to the adoption of WIoMT devices is their efficiency in saving time and effort for medical attention or health monitoring. Therefore, consideration must be given to data privacy and security. This study also narrowed the research on wearable health technology, which is much more prevalent and known among the general public than other IoT and IoMT devices and equipment. It has developed a better understanding of the trust factor in behavioural intention to use WIoMT products through exploring security and privacy. Thus, this contributes to the body of knowledge in terms of understanding the perceptions as well as the adoption of users towards WIoMT.

### 5.2. Strengths and Limitations

This study’s strength lies in its scope, which is WIoMT technology. Many previous studies have explored models to discuss the adoption of IoT and IoMT, but this study is among the few that have stayed relevant to the present times, where wearable technologies have become a necessity for many people with chronic diseases. The study also provides insight to vendors, manufacturers, and healthcare providers in understanding advanced technology from the user’s perspective to contribute to this competitive global market. Most researchers have focused on the technological aspects of the devices rather than the user’s perspective for adopting new technology, which has been the main focus of this study. The adoption of WIoMT devices by users will benefit various groups, including the government, advertising firms, manufacturers, and healthcare professionals. It will help users and the healthcare sector develop a device that is simple to use and secure for users. Understanding the user’s perception of trust towards the WIoMT from this study will contribute to new advances in the IoMT sector, such as robotic medical equipment and remote monitoring devices. Adopting technology over conventional techniques is difficult, especially in the healthcare industry. This study will help overcome the challenges of trust, security, and privacy in the usage of wearables to assist manufacturers and the healthcare sector in developing true consumer-centric technologies.

The results of this study come with limitations. First, this is a self-reported online survey which may have resulted in information and selection biases that are common with the nature of quantitative methodologies. The second limitation is around generalisability. The participants of this study are limited to Australia; hence, the results may not be generalisable to populations of other countries, which may be very different from Australia in terms of economic, social, and cultural contexts. The study has tried to minimise this limitation by incorporating participants with international backgrounds living in Australia. Still, the country’s cultural context will influence consumer behaviour. The third limitation is that the study is primarily quantitative. Hence, only objective views have been captured. Supplementary qualitative studies might provide more insight into the utility of such devices.

Lastly, in this study, a little more than half (54%) of the variance in the outcome variable was due to the independent variables considered. However, the unexplained remaining 46% variance implies to some extent that other possible domains might influence the intention to use WIoMT devices that have been missed in the research model. This study can be a basis for further research in this area. Nonetheless, the study’s results are consistent with those of other similar studies, indicating that it will benefit a wide range of people by providing information on users’ behaviour and intentions to use WIoMT devices. It will help future researchers to work towards the better acceptance and usability of similar or advanced WIoMT devices.

## 6. Conclusion and Future Research

The main purpose of this study was to investigate the factors that led to trust and eventually to the intention to use WIoMT devices, study the power of those independent variables on the outcome variable, and explore the security risks associated with adopting the WIoMT. The independent variables/domains considered in this study were perceived usability, perceived ease of use, reliability, functionality, and perceived security and privacy, mainly extracted from the TAM model.

The study had 189 responses subjected to quantitative analyses using SPSS version 21. First, the descriptive data were analysed, which showed more participants belonging to the age group of 25 to 34 years, more females, more with an above-average level of technical proficiency in using computers, more believing technology to be significant in one’s life, more familiar with WIoMT devices, and more using such devices among those who knew about such devices. Next, to check the reliability and validity of the study instrument, the internal consistency was calculated within each dimension and each domain. The Cronbach’s alpha value, which measures internal consistency, was higher than the acceptable cut-off of 0.7. This showed that the study instrument was reliable and valid in accomplishing the research objectives.

The collected data were further subjected to correlation analysis, which examined correlation coefficients among various domains and sub-domains and, thus, bivariate associations of one domain with another. It was found that all independent variables were correlated univariately with the dependent variable, as all domains were found to be significantly associated with intention to use at the *p* < 0.001 level. The correlation analysis helped validate the hypotheses explored in this study. When all product- and security-related factors increase in their influence, the resulting intention to use WIoMT devices also increases.

The regression analysis conducted also helped determine the factors with the strongest relevance when adopting WIoMT. Only three factors, i.e., perceived security and privacy, perceived usability, and perceived ease of use, were found to have significantly predicted the outcome variable (intention to use). This finding is not merely by chance but rather a statistical backup. It means that if the user perceives that that device maintains security and privacy, if it is usable and easy to use, only then would they have intention to use.

The highest power to predict the dependent variable lay within the perceived security and privacy domain. The perceived security risks were unauthorised access to data, malware infection and vulnerabilities, lack of regulation and compliance, unsecured network connectivity, lack of encryption, and lack of patching and device updates. The sub-domain of these risks was significantly correlated with intention to use. This means that if the users perceive these risks to be present in WIoMT devices, they will have less intention to use such technology.

This study contributes to the literature regarding adopting WIoMT devices in our daily lives in which usability, ease of use, and security and privacy affect the trust of the consumer, which ultimately leads to the behavioural intention to use such devices regularly. Moreover, the study highlighted that security and privacy factors have the most power to influence the use of the WIoMT, and manufacturers, healthcare providers, and vendors need to focus on what users think if they have the vision to increase the adoption of their technology in the world. As found in the study, there are many potential consumers who are yet to be familiar with or use WIoMT devices. Future research should focus on improving the acceptability and use of comparable or upgraded WIoMT technologies. Researchers should examine more individuals who are using WIoMT devices across larger geographical locations to understand more users’ perspectives towards adoption.

## Figures and Tables

**Table 1:** Practical examples of WIoMT devices.

WIoMT Device Category	Examples
Wearable Fitness Trackers	Fitbit, Jawbone, Polar loop
Wearable ECG Monitors	Fitbit sense, Fitbit Versa 3
Wearable Blood Pressure Monitors	BPM Connect, Omron platinum
Biosensors	Vital Patch, Philips wearable biosensor
Smart Patches	Bio-patch, skin patch
Ingestible Sensors	Ingestible sensing capsules

**Table 2:** Impact of security and privacy risks.

WIOMT Security and Privacy Risks	Impact
Lack of security measures, such as encryption while transmitting data	Data shared in different domains are recorded as plain text, which leads to compromised user login details.
Sensor tracking	Potential threats such as tag cloning, side channel, physical harm, and jamming threats
Poor signal communication between medical devices such as pacemakers and insulin pumps	Users rely on such devices but are at risk of cyber attacks.
Unauthorised access	Deterioration of the effectiveness of WIoMT and adverse impact on individual’s sensitive health information
Lack of standards; limited integration between devices	Errors in communication when integration is achieved.
Lack of regulation and compliance	Exposing medical devices towards cyber attacks

**Table 3:** Demographic and other characteristics of the respondents (N = 189).

Variables	Categories	Frequency	Percent (%)
**Age Group**	18–24	70	37.0
	25–34	81	42.9
	35–44	18	9.5
	45–54	13	6.9
	Above 55	7	3.7
**Gender**	Male	88	46.6
	Female	101	53.4
**Level of technical proficiency**	Far Above Average	26	13.8
	Somewhat Above Average	85	45.0
	Average	76	40.2
	Somewhat Below Average	2	1.1
**Technology has great significance in your life**	Definitely Yes	167	88.4
	Probably Yes	21	11.1
	Might or might not	1	0.5
**Familiar with WIoMT devices**	Yes	124	65.6
	No	65	34.4
**Use WIoMT devices** **(n = 124)**	Yes	77	40.7
	No	47	24.9
**Aware of WIoMT devices (n = 77)**	Wearable Fitness Trackers	45	23.8
	Wearable ECG Monitors	6	3.2
	Wearable Blood Pressure Monitors	13	6.9
	Smart Patches	4	2.1
	None	9	4.8
**Total**		**189**	**100**

**Table 4:** Security risks of WIoMT.

Statement	Response n (%)				
	Definitely Yes	Probably Yes	Might or Might Not	Probably Not	Definitely Not
Indicate your level of agreement on the security risk of “**unauthorised access to data**” impact on Wearable Internet of Medical Things.	64 (33.9)	69 (36.5)	44 (23.2)	3 (1.6)	9 (4.8)
Indicate your level of agreement on the security risk of “**malware infections and vulnerabilities**” impact on Wearable Internet of Medical Things.	59 (31.2)	68 (36.1)	49 (25.9)	5 (2.6)	8 (4.2)
Indicate your level of agreement on the security risk relating to “**lack of regulation and compliance**” impact on Wearable Internet of Medical Things.	56 (29.6)	69 (36.5)	48 (25.4)	7 (3.7)	9 (4.8)
Indicate your level of agreement on the security risk of “**unsecured network connectivity**” impact on Wearable Internet of Medical Things.	59 (31.2)	69 (36.5)	51 (27.0)	3 (1.6)	7 (3.7)
Indicate your level of agreement on the security risk of “**lack of encryption**” impact on Wearable Internet of Medical Things.	56 (29.6)	66 (34.9)	54 (28.7)	5 (2.6)	8 (4.2)
Indicate your level of agreement on the security risk of “**lack of patching and device updates**” impact on Wearable Internet of Medical Things.	52 (27.5)	75 (39.7)	46 (24.3)	9 (4.8)	7 (3.7)

**Table 5:** Internal consistency of the domains.

**Domains**	**Cronbach’s Alpha Value**	**Cronbach’s Alpha Value Analysis**
Perceived Usefulness	0.911	Excellent
Perceived Ease of Use	0.892	Excellent
Functionality and Reliability	0.853	Excellent
Perceived Security and Privacy	0.917	Excellent
Security Risk *	0.943	Excellent
Intention to Use	0.866	Excellent

* “Security risk” is the sub-domain of “perceived security and privacy” domain.

**Table 6:** Internal consistency of dimensions.

Dimensions	Cronbach’s Alpha Value	Cronbach’s Alpha Value Analysis
Product-related factors	0.929	Excellent
Security-related factors	0.917	Excellent

**Table 7:** Correlation between functionality and perceived security and privacy.

Coefficient of Correlation I	*p*-Value	Functionality Mean	Perceived Security and Privacy Mean
0.473	0.000 *	2.67	2.29

* Correlation is significant at the 0.001 level (2-tailed).

**Table 8:** Correlation between reliability and perceived security and privacy.

Coefficient of Correlation I	*p*-Value	Reliability Mean	Perceived Security and Privacy Mean
0.531	0.000 *	2.83	2.29

* Correlation is significant at the 0.001 level (2-tailed).

**Table 9:** Correlation between perceived usefulness and perceived security and privacy.

Coefficient of Correlation I	*p*-Value	Perceived Usefulness Mean	Perceived Security and Privacy Mean
0.540	0.000 *	2.04	2.29

* Correlation is significant at the 0.001 level (2-tailed).

**Table 10:** Correlation between perceived ease of use and perceived security and privacy.

Coefficient of Correlation I	*p*-Value	Perceived Ease of Use Mean	Perceived Security and Privacy Mean
0.645	0.000 *	2.12	2.29

* Correlation is significant at the 0.001 level (2-tailed).

**Table 11:** Correlation between product- and security-related factors and intention to use.

Product- and Security-Related Factors	Correlation Coefficient (with Intention to Use)	*p*-Value
Functionality	0.371	0.000 *
Reliability	0.364	0.000 *
Perceived Usefulness	0.565	0.000 *
Perceived Ease of Use	0.631	0.000 *
Perceived Security and Privacy	0.686	0.000 *
Security Risk **	0.312	0.000 *

* Correlation is significant at the 0.001 level (2-tailed). ** “Security risk” is the sub-domain of “perceived security and privacy” domain.

**Table 12:** Regression analysis.

Model Summary ^b^									
Model	R	R Square	Adjusted R Square	Std. Error of the Estimate	Change Statistics				
					R Square Change	F Change	df1	df2	Sig. F Change
1	0.744 ^a^	0.553	0.541	0.57698	0.553	45.266	5	183	0.000

^a^ Predictors: (constant), reliability, perceived usefulness, perceived ease of use, functionality, perceived security and privacy. ^b^ Dependent variable: intention to use.

**Table 13:** ANOVA results.

ANOVA ^a^						
Model		Sum of Squares	df	Mean Square	F	Sig.
1	Regression	75.348	5	15.070	45.266	0.000 ^b^
	Residual	60.923	183	0.333		
	Total	136.270	188			

^a^ Dependent variable: intention to use. ^b^ Predictors: (constant), reliability, perceived usefulness, perceived ease of use, functionality, perceived security and privacy.

**Table 14:** Regression analysis on the intention to use WIoMT devices.

Coefficients ^a^								
Model		Unstandardised Coefficients		Standardised Coefficients	t	Sig.	Collinearity Statistics	
		B	Std. Error	Beta			Tolerance	VIF
1	(Constant)	−0.324	0.179		−1.812	0.072		
	Perceived Usefulness	0.244	0.093	0.186	2.612	0.010 *	0.482	2.075
	Perceived Ease of Use	0.280	0.097	0.221	2.875	0.005 *	0.414	2.417
	Perceived Security and Privacy	0.691	0.102	0.474	6.789	0.000 **	0.500	1.998
	Functionality	0.066	0.054	0.091	1.217	0.225	0.437	2.290
	Reliability	−0.097	0.055	−0.140	−1.768	0.079	0.391	2.557

^a^ Dependent variable: intention to use. * Significant at the 0.05 level (2-tailed). ** Significant at the 0.001 level (2-tailed).

## Data Availability

The data presented in this study are available on request from a.bello@westernsydney.edu.au. The data are not publicly available due to ethical restrictions.
